# The social health impact of Eritrean refugees on the host communities: the case of May-ayni refugee camp, Northern Ethiopia

**DOI:** 10.1186/s13104-020-05036-y

**Published:** 2020-03-27

**Authors:** Kahsay Gebrehiwet, Hailay Gebreyesus, Mebrahtu Teweldemedhin

**Affiliations:** 1 Department of Political Science and International Relations, College of Social Sciences and Languages, Aksum University, Aksum, Ethiopia; 2 Department of Public Health, College of Social Sciences and Comprehensive Specialized Hospital, Aksum University, Aksum, Ethiopia; 3 Department of Medical Laboratory Sciences, College of Social Sciences and Comprehensive Specialized Hospital, Aksum University, P.O. Box: 298, Aksum, Ethiopia

**Keywords:** Refugees, Host community, Health impacts, Ethiopia

## Abstract

**Objective:**

Migration is a contemporary global issue and the exodus of refugees may potentially posit new social health challenges in the host communities. Ethiopia is a host to the second largest refugee population in Africa. The aim of this study was to explore the health impacts by the Eritrean refugees in May-ayni camp, North West Tigrai on the host community. The research used a qualitative exploratory approach. Participants were recruited using a purposive sampling technique. The primary sources of the data were in-depth interviews of 20 key informants, and focus group discussions with 30 refugees and 30 members of the host community. Transcription and translation was done verbatim and finally thematic analysis was done using an inductive approach.

**Results:**

The findings of this research indicated that the refugees in the May-ayni camp created actual social and health threats to the members of the host communities. The socio-cultural norms of the host peoples were disrupted in terms of their social insecurity and introduction of health related challenges such as the spread of sexually transmitted infections and other reproductive health problems.

## Introduction

In 2018, the number of forcibly displaced people was estimated to be around 70.8 million, worldwide which resulted in the highest number of refugees (25.9 million) and asylum-seekers (3.5 million) ever [1]. One person in 108 people in the world today is either a refugee, internally displaced, or seeking an asylum [1]. Most refugees settle in developing countries according to the United Nations High Commission for refugees [2]. This trend in displacement reflects the conflict and turmoil faced by those in situations of severe poverty, marginalization, and insecurity. As these individuals move out in search of a better opportunity, safety, and freedom, they are seen to face new, challenging, and sometimes unwelcoming environments such as refugee camps and communities which see these newcomers as imposing food security, health, economic, environmental, and security burdens on their hosts [3].

Ethiopia has surpassed Kenya as Africa’s largest refugee-hosting country after hundreds of thousands of Eritreans, Somalis, and South Sudanese arrived in the country [4]. The total refugee population in Ethiopia has reached almost 630,000, raising concerns that its capacity to help displaced people may be overstretched. The number of Eritrean refugees coming into Ethiopia has shown a steady increase over the last several years from an average of as low as 250 to 300 a month in 2009 to an average of 2000 a month in 2014 [3]. UNHCR reported that there were over 175,000 Eritrean refugees in the Tigrai refugee camps [1]. Refugee influxes in Ethiopia are primarily due to ongoing political and civil unrest as well as recurring natural disasters in neighboring countries. Currently there are a total of nine refugee camps located in the East, West, and Northern parts of the Ethiopia [5]. Among these, four refugee camps are located in the Western and North-western zone of Tigray region [3]. With very limited access to land and livestock, refugees have been forced to survive on meager resources. For instance, in the case of Shimelba camp, land is especially a problem as the camp is located near the town of Sheraro, which is very close to the border of Eritrea. As is the case with other camps, refugees receive food and other assistance through the World Food Program (WFP), Administration for Refugee and Returnee Affairs (ARRA), and UNHCR.

Studies conducted in other African countries such as Kenya and Uganda, reported that the conflicts between the refugee and the host community explained by security, resource shortages, and socio-cultural factors had complicated the livelihood of the host communities [6–8]. In Ethiopia, some research has been done regarding this issue particularly in Gamblla and Beni-shungul Gumuz region but not in Tigray Region. Moreover, only a few studies have addressed the impact of refugees on their host population, focused on the economic and security aspects [9]. To the researcher’s knowledge, the impact of the presence of refugees on social health of the hosting community has not been extensively explored, particularly in May-ayni refugee camp.

## Main text

### Methodology

#### Study area, study period and design

A qualitative study was conducted at May-ayni refugee camp from September 2018 to May 2019. May-ayni refugee camp is located in the North West Zone of Tigray, 1093 km from Addis Ababa. The camp has a total of 15,000 Eritrean refuges. The hosting community has a total population of 36,000, which is served by one public health center and two health posts.

### Sample and sampling procedure

The researchers used purposive sampling to select 20 key informants. The key informants were comprised of health care workers (3), coordinators of the local NGOs (3), representatives from the security and community police (4), women association representatives (2), youth association representatives (2), local administrative workers (4), and religious leaders (2). Based on the total number of households in the refugee camp and nearby villages as a sampling frame, the researchers randomly selected 30 respondents from the host community and 30 respondents from the refugee camp. Hence, a total of 80 respondents have participated in the study. Therefore, the study included diverse participants to generate rich information through focus group discussions and in-depth interviews.

### Data collection procedures

This research used primary data sources, including refugees, donor agencies, charitable organizations, and host communities as well as local inhabitants who live proximal to the refugee camp. The data was collected via ten focus group discussions with the randomly selected respondents from the refugees and the host community and augmented by in-depth interviews with the purposively selected 20 key-informants.

### Data analysis

This study is descriptive, narrative and analytical. It explored contextually analyzed and presented the recent impacts of refugees on the social health of a host community. The audio-taped interviews, focus group discussions, and field notes were transcribed and translated verbatim into English, and imported into ATLAS.ti7 for Windows™ for coding and analysis. The coded data was categorized under suitable themes and sub-themes using as inductive approach as presented in the results.

### Results

#### Refugees’ living conditions and host community

According to the respondents from the host community, there have been many co-operative activities for the wellbeing of both refugees and host community members (Table 1). For example, a joint committee for mutual discussion, sharing of social services and related issues exists. Conversely, the presence of the refugee camp had impacts on the host community according to host community respondents who described environmental, socio-economic, and security impacts. However, refugee respondents did not consider these impacts but rather spoke of their intention not to stay but to get to their destination through the involved NGOs. Thus, the main concern of refugees regarding their life in the camp is to get to their destination (another country), while host community members were worried about the long term impact on their socio-economic life due to the proximity and size of the refugee community. According to the respondents:

“*I just came here not to live here in Ethiopia but to go to some of the European countries by the help of the international NGOs in the refugee camps. I thought this is the best camp to get the support from NGOs. However, I am having very difficult living conditions for the past 3* *years*” (A 28 year old male).

“*…we get support such as water source, electricity and health care from the host community and local NGOs but the camp is very crowded. Additionally, some refugee members commit crimes inside the camp and in the nearby towns. Therefore, the living condition here is not safe*” (A 20 years old female refugee).

“*…the ultimate goal of the Eritrean refugees is not to sustain their life here around but to move to some other developed countries. Hence, they misuse the resources of the host community; the crowded living condition in the camps is also contributing for the undesirable wastage of resources and misconduct*” *(A 48* *year old respondent from the community)*

**Table 1:** Socio-demographic characteristics of the study participants

Characteristic	Number (%)
Age	
18–24	19 (23.7)
25–29	47 (58.8)
30–49	14 (17.5)
Total	80 (100)
Gender	
Male	58 (72.5)
Female	22 (27.5)
Total	80 (100)
Participant category	
Refugee	30 (37.5)
Host community	30 (37.5)
Key-informant	20 (25)
Total	80 (100)
Occupation	
Unemployed	30 (37.5)
Employed	27 (33.8)
Merchant	17 (21.2)
Farmer	6 (7.5)
Total	80 (100)

#### Refugees’ livelihood contribute to health and social insecurity of host community

In the study area, refugee impact on health and social aspects negatively affected to the host community. According to the respondents they have suffered by various social and health related issues, such as sexual transmitted diseases, social discrimination in the case of females are trying to marry with refugee, prostitution, drug addictions, and other related issues. There are a large number of youth refugees in the camp; hence, the surrounding host community is increasingly vulnerable to various sexual transmitted diseases. Further, the respondents felt that it is becoming a social crisis as local females are assumed to have contact with the refugees which label them as at risk for these diseases. This is summarized in the following quotations:

“*…we refugees are jobless and dispirited here. We are socially and economically dependent on the host community and of course, the host community is highly affected in terms of resources, security and communicable disease*” (A 19 year old refugee male).

“*The expansion of sexual transmitted disease has been increasing from time to time particularly from the establishment of the refugee camps. The adolescents in the refugees try to attract poor ladies from the hosting community by giving money or by intimidation which leads to multiple sexual relationships*” (A 42 year old male respondent from the key informants).

“*The youths in the refugee are mostly hopeless and they are addicted to alcohol and cigarette smoking. They spend much of their time in the nearby night clubs; they invite adolescent females from the hosting community for sexual relationships and expose them for STI and other reproductive health problems such as unintended pregnancies and septic abortion*” (A 35 year old female respondent form the community).

#### Social discrimination on female host communities

According to the respondents, there are many problems related with reproductive and sexual harassment. The following indicate the insights from the key informants:

“*When youth from the refugee camp make sexual contact with any member of the host community, she is totally discriminated by almost the host community. This perception and attitudes within the community impose on females that are vulnerable to the problem which they are pushed to wrong decision such as abortion and taking wrong drugs*” (A 40 year old female).

“*Females from the host communities start sexual relationships with youths from the camps assuming that they will go together to some other developed countries by the help of the NGOs. Gradually, they grow children without valid marriage and the females become the victims of social discrimination. On the other hand, only a few succeed to establish stable marriage with refugees*” (A 26 year old male).

“*From my experience, most of the females who had sexual contact with the youths of the refugee camps are HIV/AIDS carriers. As a result they assume themselves as socially discriminated. To escape from the social discrimination, they committed different activities including suicide attempts and some are disappearing from the community*” (A 32 year old female respondent).

### Discussion

Migration is rapidly becoming one of the most critical global health challenges. Predominantly persecution, conflict, generalized violence and human rights violations are the major issues that escalate displacement and migration [2]. As the countless refuges cross into neighboring countries they face complicated life experiences and challenges as the move from their home to often unwilling or under-prepared hosting states. Basically, refugees or displaced peoples are forced to seek the minimal level of goods and services for mere survival, even depending or usurping on host community resources and properties. In addition, refugees impose direct and indirect threats to their host communities due to humanitarian issues, and the heavy requirements for basic material resources [10]. This study revealed that there are perception of key informants that the refugees have long term health impacts on hosting communities, primarily related to the issues of sexual harassment, sexual abuse, and expansion of sexually transmitted infections in the host community. The influx of sexually active adolescents in the refugee camps has led to increased demand of social interaction with females in the host community. However, the risky behaviors of the dispirited members of the refugees can lead to sexual violence, unintended pregnancies, and increased predisposition to STI such as HIV. This emerging reality pose long-term social and mental health impacts on members of the host community [11]. This qualitative finding refutes what has been suggested in a previous study which failed to establish sufficient connection between refugees, wide-scale rape, and increased prevalence of HIV on the host communities [12]. However, the finding of the current study is consistent with a study conducted among Somali refugees in Ethiopia though the focus was inside the camps only [13].

As compared to non-refugees, refugees have low quality of life due to their educational and occupational status [14]. Hence, as the camp expands there is increased competition, criminality, and violence related to resources between the host community and refugees [15], resulting in physical trauma and other mental health problems as revealed in the current study. Studies had also indicated that there is high prevalence of depression and substance use in refugees due to post-migration socio-economic factors [6, 16]. According to a previous study conducted among Eritrean refugees, pre-migration and post migration living difficulties were directly associated with depressive symptoms [17] which may contribute to substance abuse, polyamorous behaviors, and criminal activity (including sexual abuse). Some have suggested the need for programmatic intervention packages to reduce violence and the social health impact of refugees to the host community [18]. Interventions on improving social support, management of emotions, and task oriented coping styles may be important to reduce depression and other risky behaviors among refugees [18].

### Conclusion

This study has explored the impacts of refugee on host communities through social and health perspectives. The findings of this research indicated that there are perceptions that the refugees found in the May-ayni camp in Tigray Ethiopia posits actual social-health threats to the hosting communities. It was evident that the host community’s key informants felt the impacts were primarily in the area of security (violence, crime) and sexual behaviors that were outside the social norms of the community.

## Limitations

The study was conducted in one refugee camp; inference of the findings for all of the refugee camps is highly limited.

## Data Availability

The data set used and/or analyzed during the current study is available from the corresponding author on reasonable request.

## Abbreviations

ARRA: Agency for Refugees and Returnees Affair
NGO: Non-governmental organizations
STI: Sexually transmitted infections
UNHCR: United Nations Higher Commissioner for Refugees
