# Correction to: The social health impact of Eritrean refugees on the host communities: the case of May-ayni refugee camp, Northern Ethiopia

**DOI:** 10.1186/s13104-020-05414-6

**Published:** 2021-01-25

**Authors:** Kahsay Gebrehiwet, Fitsum Abrha, Hailay Gebreyesus, Mebrahtu Teweldemedhin

**Affiliations:** 1 Department of Political Science and International Relations, College of Social Sciences and Languages, Aksum University, Aksum, Ethiopia; 2 Department of Civics and Ethical Studies, College of Social Sciences and Languages, Aksum University, Aksum, Ethiopia; 3 Department of Public Health, College of Social Sciences and Comprehensive Specialized Hospital, Aksum University, Aksum, Ethiopia; 4 Department of Medical Laboratory Sciences, College of Social Sciences and Comprehensive Specialized Hospital, Aksum University, P.O. Box: 298, Aksum, Ethiopia

## Correction to: BMC Res Notes (2020) 13:182 https://doi.org/10.1186/s13104-020-05036-y

Following the publication of the original article [1] we were informed that author Fitsum Abrha had unfortunately been omitted from the author list, as he was unable to respond during the manuscript preparation and submission.

The authorship has now been updated in the original article and included in this Correction.

The ‘Author contributions’ section has also been updated to read: “KG and FA: conceived and designed the project; managed the data collection. KG, HG and MT: conducted data analysis and wrote the manuscript. All authors read and approved the final manuscript”.
